# The use of cardiac magnetic resonance imaging in diagnosing and characterizing hypertrophic cardiomyopathy

**DOI:** 10.1186/1532-429X-18-S1-P168

**Published:** 2016-01-27

**Authors:** Emmanuel Ogele, Lubna Choudhury, James C Carr, Jeremy D Collins

**Affiliations:** Feinberg School of Medicine, Northwestern University, Chicago, IL USA

## Background

In Hypertrophic Cardiomyopathy (HCM), measurements of maximal myocardial thickness serve a critical role in diagnosis and risk stratification. CMR plays an increasingly significant clinical role. Maximum wall thickness is commonly measured at both MRI and TTE often with discrepancies. The purpose of this study is to examine the influence of slice obliquity on CMR-measured maximum myocardial thickness in the basal anteroseptum comparing 3-Chamber long-axis with short-axis CMR imaging and further, to assess the degree of agreement between long-axis measurements on TTE and MRI.

## Methods

We performed a retrospective analysis of 50 consecutive patients (29 males and 21 females; age 58 ± 14) referred for CMR at Northwestern Memorial Hospital with asymmetric septal HCM. The 3-chamber (3Ch) balanced steady state free precession (bSSFP) images were cross-referenced with the short axis (SA) bSSFP cine stack to ensure correlation between measured myocardial regions. The basal anteroseptum was measured on the 3-chamber images in diastole using a line parallel with the mitral valve and on the short axis view, a line orthogonal to the myocardium at the region corresponding to that measured in the 3 chamber view. The angulation in degrees between the 3-chamber bSSFP slice position and the orthogonal measurement on short-axis images was recorded. In cases with significant discrepancy between 3Ch and SA, measurements of the basal anteroseptum were performed on parasternal long axis (PSL) images at TTE. Measurements were compared using the student's t-test, with a p-value of 0.05 considered significant.

## Results

**Figure 1:**
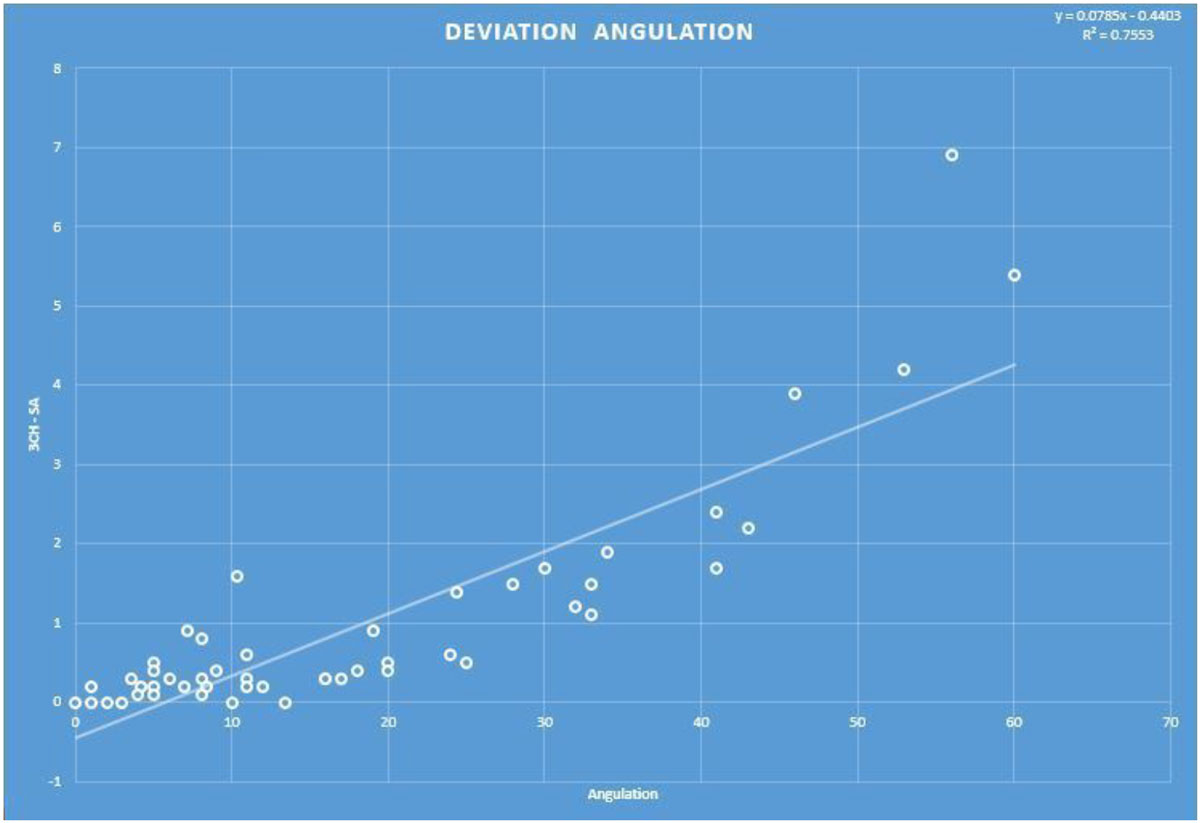
**The value of the difference between the measurements obtained on 3-Chamber and Short axis imaging plotted against angulation**.

On aggregate, there was a difference between CMR derived 3Ch and SA myocardial thickness (p<.0005) with an average difference of 0.99 ± 1.4 mm. Angulation between 3Ch and SA images averaged 18 ± 15°. There was a positive linear correlation between angulation and the difference noted between CMR derived 3Ch and SA measurements (R^2^ = .755, p = .012. See Figure 1).12 subjects (24%) demonstrated at least a 1.5 mm difference between CMR derived 3Ch and SA measurements corresponding to an angulation of 24.7°. 4 subject were reclassified below HCM diagnostic threshold after SA measurement. TTE measurements showed a close agreement with 3Ch measurements (mean TTE-3Ch = .85 mm) while tending to overestimate the short axis measurements (mean TTE-SA = 1.69 mm).

**Figure 2:**
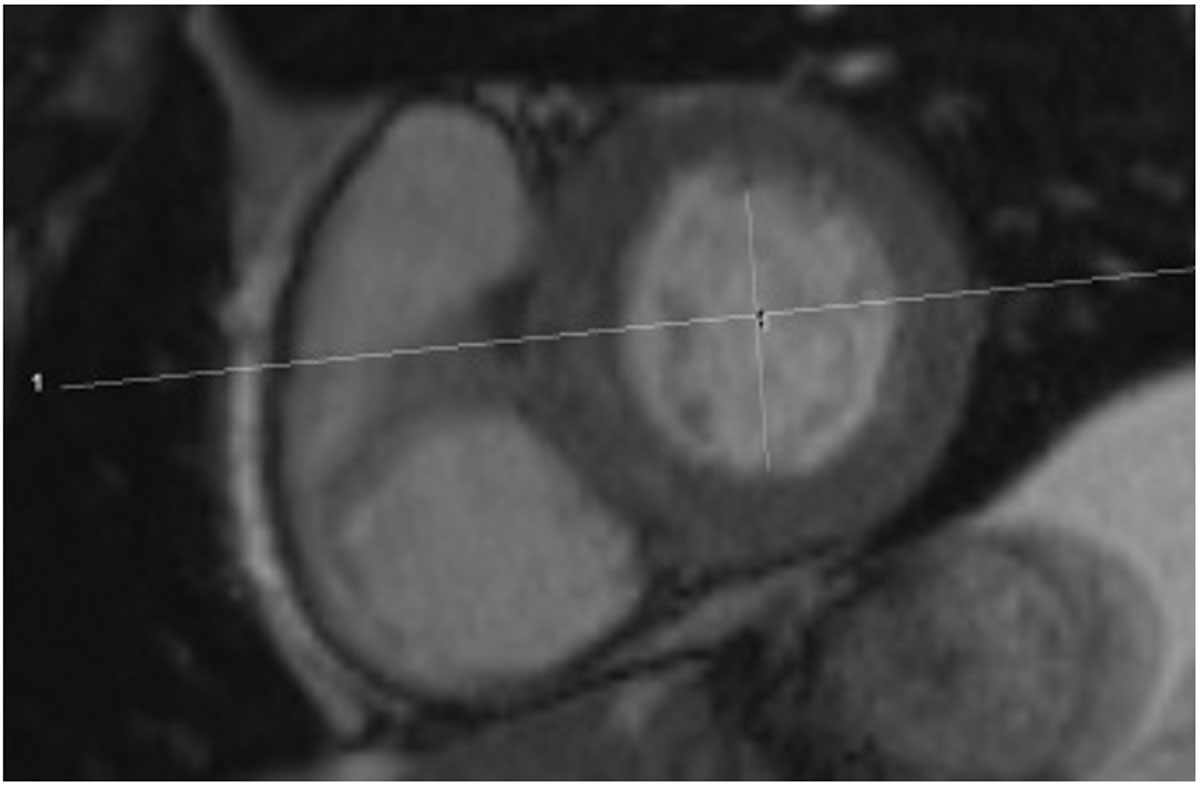
**Orthogonal planning of the 3 Chamber view as cross referenced to the Short axis view**.

**Figure 3:**
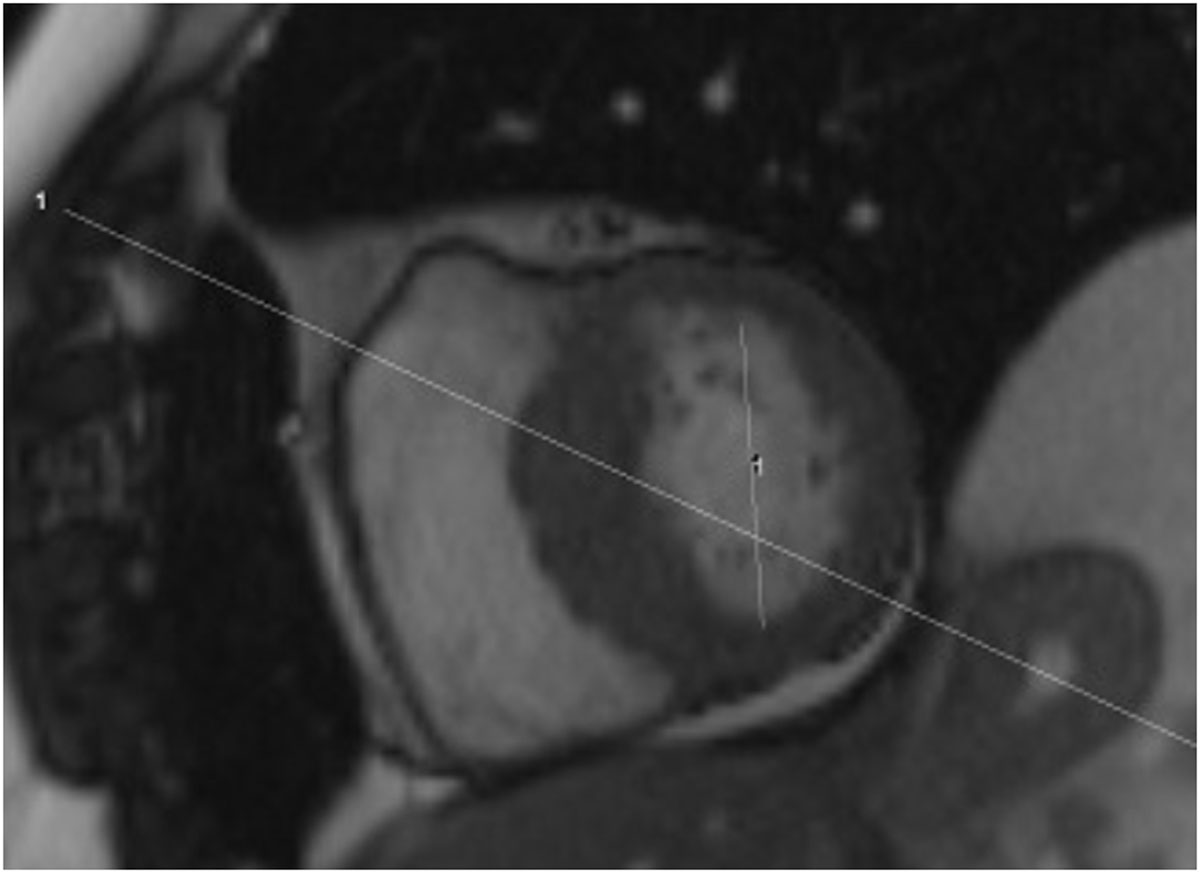
**Non-orthogonal (oblique) planning of the 3 Chamber view as cross referenced to the Short axis view**.

## Conclusions

Angulation of long-axis imaging may explain differences between long-axis and short-axis measurements. Although the absolute difference averaged 0.99 mm in this study, the reliance on maximal myocardial thickness in the diagnosis of HCM is potentially significant, with nearly ¼ of subjects in our study demonstrating differences greater than 1.5 mm. 3Ch images agreed more with TTE measurements suggesting similar challenges with angulation. Our results suggest that operators should minimize 3Ch angulation; keeping the angulation to < 20 as this corresponds to a difference of 1 mm and is within the expected error of repeat measurement.

